# Gating Gas Permeability Through Dynamic Cracking of Liquid Crystal Polymer Membranes

**DOI:** 10.1002/smll.202503444

**Published:** 2025-06-16

**Authors:** Yuxin You, Youssef M. Golestani, Mert O. Astam, Danqing Liu

**Affiliations:** ^1^ Human Interactive Materials (HIM) Department of Chemical Engineering and Chemistry Eindhoven University of Technology Groene Loper 3 Eindhoven 5612AE The Netherlands; ^2^ Institute for Complex Molecular Systems (ICMS) Eindhoven University of Technology Groene Loper 3 Eindhoven 5612AE The Netherlands

**Keywords:** dynamic cracking, gas permeability regulation, hybrid bilayer membrane, liquid crystal oligomer networks, stimuli‐responsive polymers

## Abstract

Intelligent membranes promise transformative advances in real‐time control of substance permeation, surpassing current technologies through their intrinsic adaptability to environmental stimuli. In this work, a material‐regulated approach to dynamically control substance permeation, such as gas, using hybrid bilayer membranes composed of gold‐coated liquid crystal oligomer networks (Au‐LCONs), is established. Thermally driven LCON actuation induces a stress mismatch at the LCON‐Au interface that cracks the Au layer, effectively opening “gates” in the impermeable Au to allow gas transport through the membrane; this reversible effect can be precisely controlled with temperature, facilitating the use of this system for triggering gas‐mediated chemical reactions on demand. Furthermore, switchable gas transport can be localized by the patterned Au coating on LCONs, restricting gas flow and chemical reactions to designated areas. This work paves the way for advancing intelligent materials for applications with precise and switchable substance permeability requirements, such as environmental monitoring, drug delivery, preservation systems, and filtration technologies.

## Graphical abstract

A stimuli‐responsive polymer membrane enables reversible and localized control of gas transport. Integrating a passive gold layer and an active liquid crystal oligomer network (Au–LCON), heat‐triggered actuation induces interfacial stress mismatches that dynamically open or close cracks, which serve as gas channels. The membrane also functions as a chemical valve, facilitating gas‐mediated reactions for on‐demand functionalities.

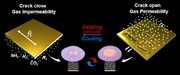

## 1 Introduction

Dynamic control of substance exchange is crucial in nature, demonstrated by the sophisticated mechanisms developed within various organisms to regulate substance permeation to adapt to changing environmental conditions. The cacti are a prime example among drought‐enduring plants, exhibiting an advanced strategy of water conservation through the regulated opening and closing of stomata on their leaves.^[^ ^1^, ^2^ ^]^ Similarly, beetles showcase a finely tuned tracheal system for gas regulation, adjusting their spiracles to balance oxygen intake and carbon dioxide release.^[^ ^3^, ^4^ ^]^ In more complex organisms, such as mammals, cell membranes regulate the transport of ions, metabolites, and other specific molecules through protein channels for homeostasis.^[^ ^5^, ^6^, ^7^ ^]^ The core method that lies at the heart of these natural examples is the “gating” of membranes, dynamically modulating permeability to control the exchange of substances.^[^ ^8^, ^9^, ^10^, ^11^, ^12^ ^]^ In our work, we are inspired by these biological membranes to create an intelligent membrane using a hybrid responsive organic‐passive inorganic bilayer system. Triggered by the thermally driven actuation of the responsive layer, our system possesses a dynamic “gating” functionality that “opens” and “closes” substance permeability, such as gas, through control of cracking in the passive layer.

**Figure 1:**
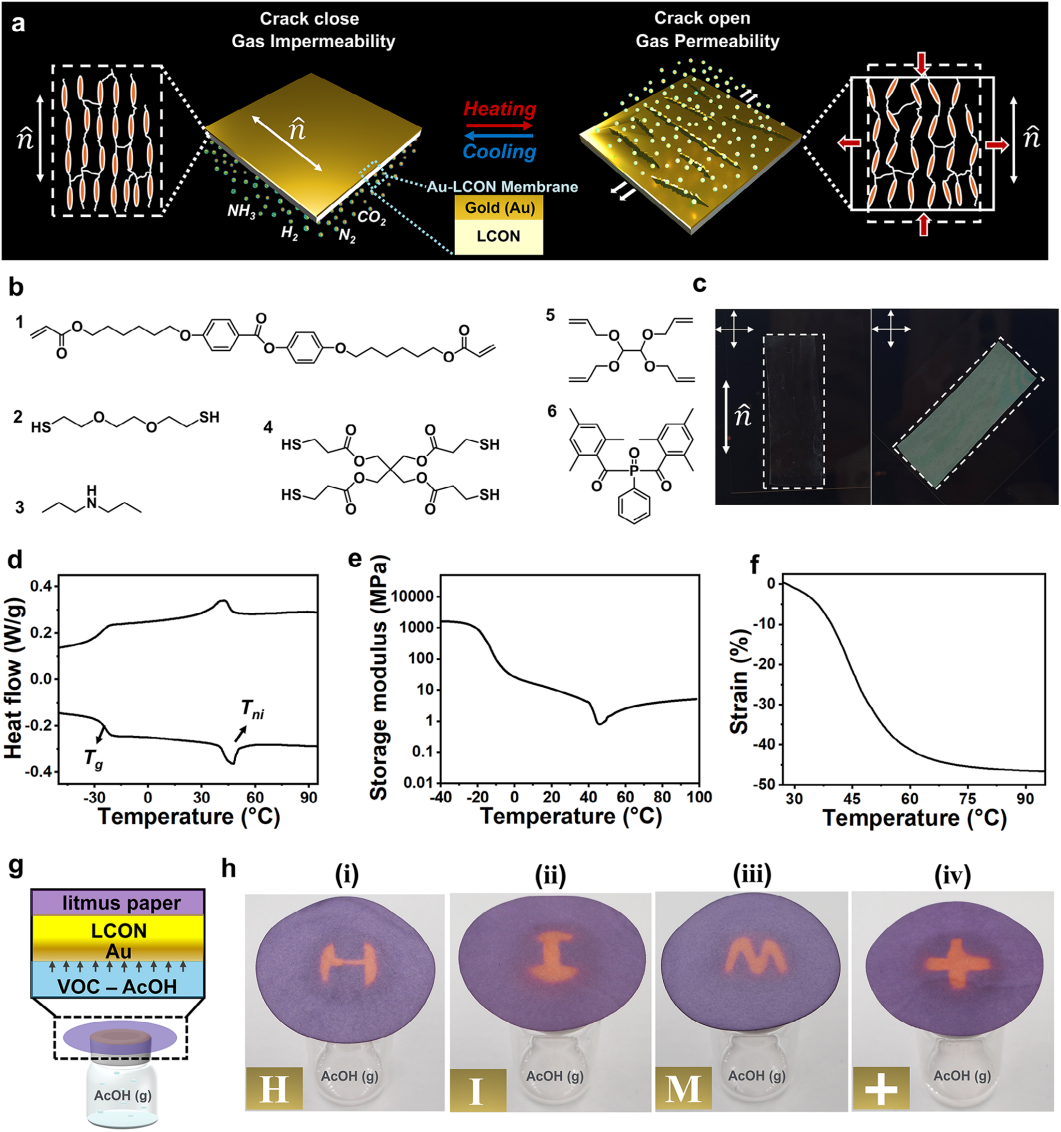
Design strategy of the dynamic Au‐LCON membranes for switchable gas permeability. a) Schematic illustration of the switchable gas permeability mechanism; annotations display the corresponding liquid crystal alignment and material structure in the permeable and impermeable states. b) Structural formulae of the chemical reagents involved in the synthesis of LCON material. c) Polarized optical images confirm the uniaxial alignment of the LCON; $\hat{n}$ denotes the director of the alignment, and the polarizing directions of the crossed‐polarizers are marked. d) Graphical representation of results from differential scanning calorimetry measurements, marked with the glass transition (*T*_g_) and the nematic‐to‐isotropic phase transition (*T*_ni_) of the LCON. e) Graphical representation of the changes in storage modulus and f) actuation strain of the Au‐LCON with temperature. g) Schematic illustration of a device for visualizing gas permeation with volatile organic compounds (VOC)‐AcOH. h) Images of localized permeation of AcOH leading to region‐restricted purple‐to‐red color changes on the litmus paper; the inserts in the bottom left represent the mask designs corresponding to each patterned Au‐coated region in (i)–(iv).

We achieve this hybrid bilayer membrane design through our gold‐coated liquid crystal oligomer network (Au‐LCON) configuration (**Figure** **1**a), where the LCON serves as the soft‐elastic driving layer, which undergoes phase‐transition‐induced anisotropic deformations in response to thermal stimuli.^[^ ^13^, ^14^, ^15^, ^16^, ^17^, ^18^, ^19^, ^20^, ^21^, ^22^, ^23^ ^]^ Intrinsically, our LCON materials are gas‐permeable due to their loosely crosslinked structure,^[^ ^24^, ^25^, ^26^, ^27^, ^28^, ^29^, ^30^ ^]^ while the Au coating is impermeable, limiting the diffusion of substances due to its dense atomic packing; thus, the Au functions as a brittle hard barrier and the system is initially non‐permeable.^[^ ^31^, ^32^, ^33^ ^]^ Yet, when thermally activated, the LCON undergoes anisotropic deformations, contracting along and expanding perpendicular to its molecular orientation, typically labeled as the director ($\hat{n}$), which generates stresses at the LCON‐Au interface. When a critical threshold stress is exceeded, cracks develop on the Au layer, allowing gas to permeate; nonetheless, this action is reversible and, once the driving thermal stimuli are removed, the LCON can relax to its original shape and close the cracks in the Au layer, rendering the membrane impermeable again. We incorporate our Au‐LCON membranes as a chemical valve to enable on‐demand triggering of chemical reactions in devices through switchable gas permeability. We also design localized functions of our Au‐LCON membranes through patterned Au coating techniques. Therefore, we believe the dynamic substance exchange capabilities of our intelligent membranes promise great impact in applications ranging from environmental filtration to medical devices and fluidic controlled‐release systems.

## 2 Results and Discussion

We fabricate our Au‐LCON intelligent membranes through the two‐step polymerization process, where our reagents involve a diacrylate mesogen (**1**), flexible dithiol spacer (**2**), catalyst (**3**), tetra‐functional thiol‐ (**4**) and ene‐(**5**) crosslinkers, and photoinitiator (**6**) (Figure 1b). Mixing these reagents triggers a Michael addition reaction, forming a loosely crosslinked liquid crystal oligomer film; we optimize a thiol‐to‐acrylate molar ratio of 1.2 to induce the presence of thiol functional groups by the end of the first curing step, ensuring that a secondary thiol‐ene photo‐polymerization step is possible. The resulting free‐standing film is mechanically stretched to a strain of 100% to align liquid crystal mesogens into a uniaxial structure, which is then arrested by activating secondary thiol‐ene *in‐situ* photo‐polymerization using UV irradiation and photoinitiator; we confirm the alignment by analysis of our LCONs between crossed polarizers (Figure 1c). To establish our bilayer configuration, we deposit a metallic Au layer on top of the LCON through sputter‐coating techniques. Au strongly adheres to our LCONs due to the strong affinity between Au and sulfur,^[^ ^34^, ^35^, ^36^ ^]^ commonly attributed to the formation of Au‐thiolate bonds, where sulfur atoms in the thiol groups donate a lone pair of electrons to form stable covalent bonds with the Au atoms. Yet, recent studies suggest that the Au–sulfur interfacial coupling may occur through a physiosorbed interaction instead.^[^ ^37^ ^]^ As for the thermal properties of the LCON, its nematic‐to‐isotropic phase transition can be set to initiate at 40 °C (Figure 1d), programming the membrane to be stable at ambient conditions and activate when temperatures are increased above room temperature; this behavior is selected with applications requiring stringent temperature control in mind, such as pharmaceutical preservation.^[^ ^38^, ^39^ ^]^ Since the interaction between our LCON material and Au coating is stress‐based, we measure the storage modulus (Figure 1e) and the actuation strain (Figure 1f) of the Au‐LCON bilayer at various temperatures and compare it to that of pure LCON (Figure S1, Supporting Information). We observe that Au‐LCON maintains the same modulus as pure LCON and also preserves a comparably high actuation strain, indicating that the addition of the Au layer does not adversely affect the membrane's mechanical performance in a significant manner.

To demonstrate the gas permeability of the LCON and the impermeability of Au, we design a device to visualize these properties, which consists of a chamber containing acetic acid (AcOH) vapor that is sealed off with our membrane; a purple litmus paper is placed above the membrane (Figure 1g). The gas permeation process involves the adsorption of AcOH, followed by diffusion of AcOH inside, ultimately, desorption of AcOH from the membrane.^[^ ^40^, ^41^ ^]^ By patterning Au coatings on the LCON through the use of masks in the sputter‐coating process, localized gas permeable functionality in selected regions on the LCON is unlocked; at the Au‐coated areas, AcOH is adsorbed only onto the Au surface and cannot penetrate it. On the other hand, in the uncoated regions, AcOH is able to diffuse into the LCON layer and is subsequently desorbed from the top surface. Once AcOH reaches the litmus paper, it undergoes a color change from purple to red that indicates hydrogen ions have reacted with the contained dye; by demonstrating that this phenomenon can be induced in selected regions expressly, we confirm localized gas permeation (Figure 1h). Yet, this process is slow, with gradual red intensification over time on the litmus paper, described in detail in Figure S2 (Supporting Information).

**Figure 2:**
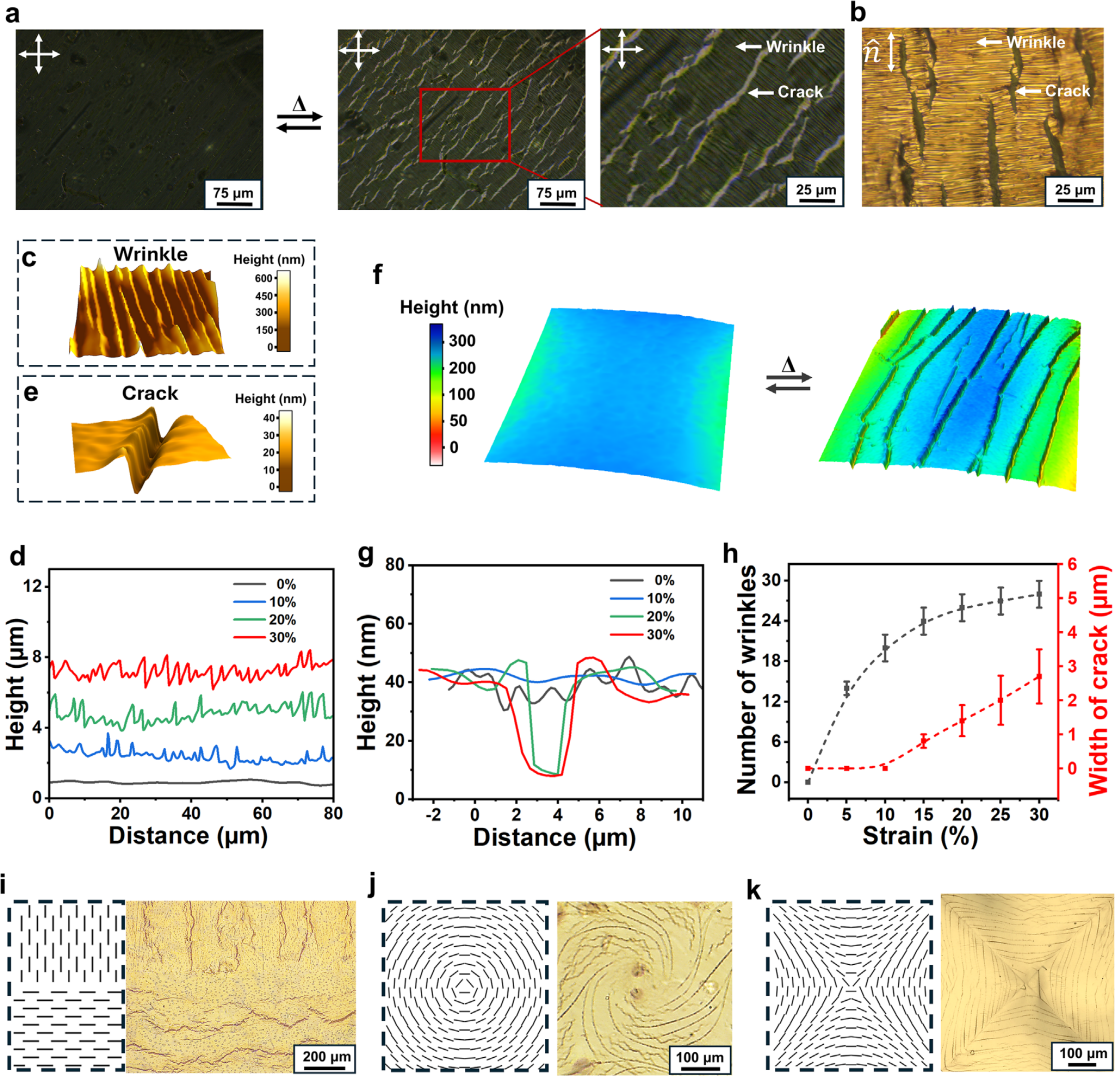
Formation of the dynamic cracks in the Au‐layer of the Au‐LCON membrane. a) Annotated transmission POM images and b) reflective optical microscopy image of the reversible wrinkle and crack formation in the Au‐layer with the application and removal of thermal stimuli; the scale bar and polarizing directions of the crossed‐polarizers are marked. c) 3D visualization of wrinkles on the Au‐layer. d) Graphical representation of the cross‐sectional spatial profiles of the wrinkles in the Au‐layer at different LCON strains. e) 3D visualization of an individual crack in the Au‐layer, reconstructed from data collected through AFM. f) Interferometer data reconstruction of the Au‐layer undergoing reversible crack opening with thermal stimulus. g) Graphical representation of the cross‐sectional profiles of the cracks in the Au layer at different LCON strains. h) Scatter plot of the number of wrinkles and the width of cracks in the Au‐layer as a function of strain. Schematic representation and corresponding reflective optical microscopy images of cracking in the Au‐layer of LCONs with i) adjacent line domains with orthogonal directors, j) +1 azimuthal alignments and k) −1 azimuthal alignments.

Building on the Au‐LCON gas permeability principle, we thermally activate our uniaxially aligned Au‐LCON membranes to investigate their dynamic “gating” functionality; as the temperature gradually increases, the Au coating first wrinkles, as observed under the optical microscope (**Figure** **2a,b**) and interferometer (Figure 2c). The wrinkling arises from a stress mismatch between Au and the LCON interface during thermal contraction along the $\hat{n}$ of the LCON, resulting in the generation of wrinkles perpendicular to $\hat{n}$; we witness that wrinkling intensifies and becomes denser with increasing temperature, caused primarily by greater LCON contractions along $\hat{n}$ (Figure 2d). During this process, the LCON undergoes a larger anisotropic deformation, producing a larger strain. The deformation continues until it reaches a critical strain of ≈12% at temperatures of ≈40 °C (Figure 1f) where cracks appear, as observed with atomic force microscopy (AFM) (Figure 2e) and the interferometer (Figure 2f). At this stage, the in‐plane stretching strain at the Au‐LCON interface is large enough to cause the Au layer to crack which propagates along the $\hat{n}$ (Figure 2b,e,f). The cracked regions appear bright under the transmission polarized optical microscope (POM), due to the underlying LCON layer, as identified in Figure 2a. The width of the cracks expands as the deformation of the LCON continues to increase (Figure 2g). We note that the number of wrinkles and the width of cracks on the surface of the membrane do not follow the same trend. Specifically, the population of wrinkles increases with the amount of strain, and as the strain further increases, the wrinkling reaches a saturation state, where the number of wrinkles stabilizes without significant further increase^[^ ^42^, ^43^ ^]^; while, there is a threshold strain needed to initiate cracks, beyond which the cracked area continues to grow with increasing strain (Figure 2h). Furthermore, we observed that greater thickness of the Au layer led to smaller cracked areas, referred to as crack density (CD), as shown in Figure S3 (Supporting Information); this can be attributed to the increased fracture toughness of thicker Au layers,^[^ ^44^ ^]^ which allows for a more uniform distribution of applied stresses. Yet, the effects of the cracking process are reversible, as cooling to room temperature allows the LCON to return to its original state, withdrawing the force exerted on the Au layer and subsequently allowing the cracks to close.

Extending the functionality of our membranes beyond uniaxially aligned Au‐LCONs, we also achieve complex dynamic crack patterns using LCONs with patterned alignments. For example, adjacent line domains with orthogonal $\hat{n}$ (Figure 2i), +1 azimuthal alignment (Figure 2j), as well as ‐1 azimuthal alignment (Figure 2k) are established. The detailed fabrication process of patterned LCON is shown in Experimental Section. Upon actuation, we find that, in all cases, cracks in the Au layer propagate along the orientation of $\hat{n}$. The LCON, aligned in adjacent line domains with an orthogonal $\hat{n}$, forms orthogonal cracks (Figure 2i), and the +1 azimuthal alignment results in circular cracks (Figure 2j), while the ‐1 azimuthal alignment generates cracks in a curved rhomboidal pattern (Figure 2k). This is a testament to the varied functionalities and design freedom offered by the use of responsive LCON materials in intelligent membrane design.

**Figure 3:**
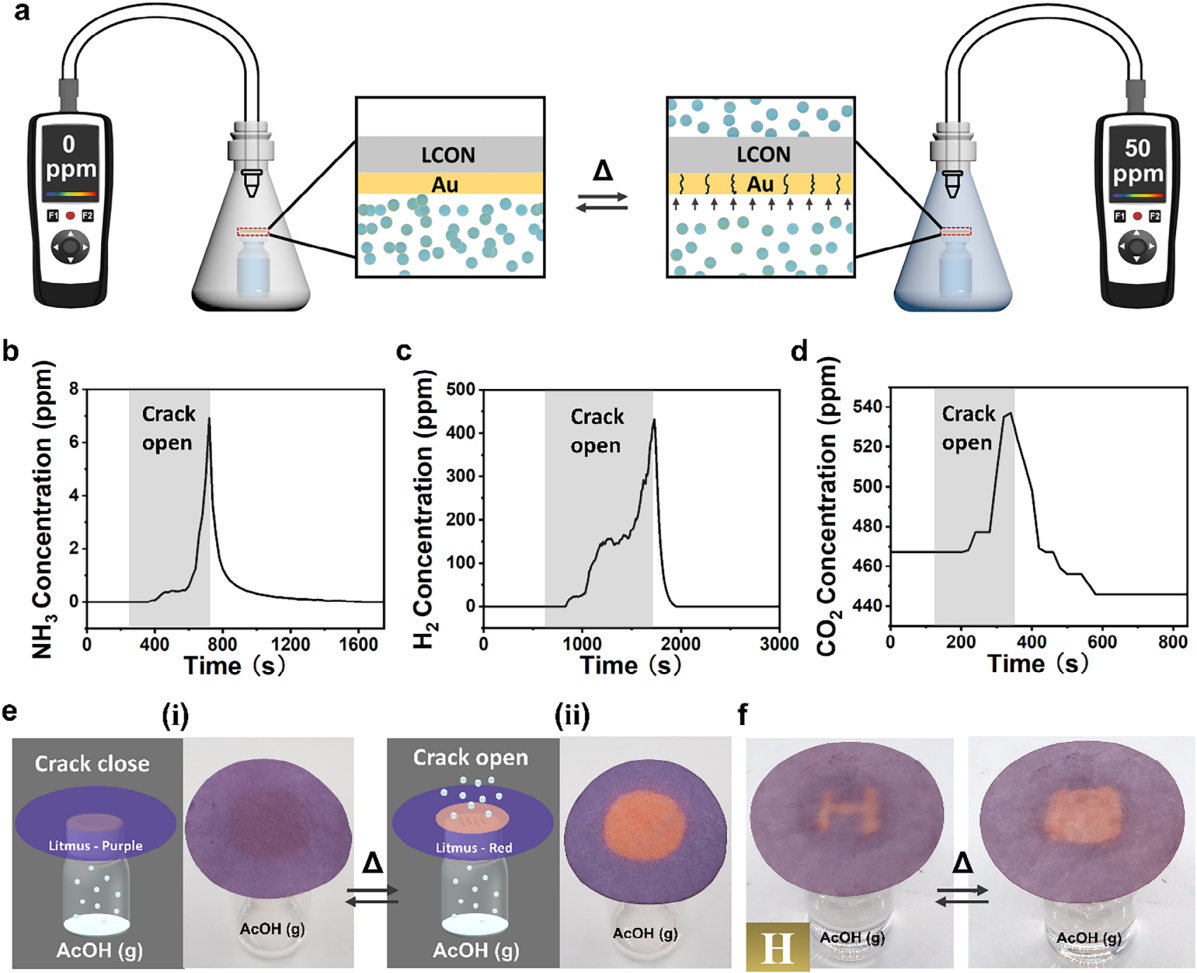
On‐demand switching of Au‐LCON gas permeability through dynamic crack opening and closing. a) Schematic illustration of the Au‐LCON gas permeation measurement and its thermally driven change in gas permeability due to crack formation; gas particles are marked in blue. Graphical representation of b) NH_3_, c) H_2_, and d) CO_2_ concentration over time; the times of the opening of Au‐layer cracks are marked, followed by the closing of these cracks. e) Schematic illustration and corresponding images of the visualized AcOH permeation test on our Au‐LCON membranes when cracks are i) closed and ii) open. f) Images of the passive‐active localized permeation of AcOH with thermal stimuli, enabled through the patterning of Au‐coating on Au‐LCON membranes.

We gauge the performance of dynamic‐crack‐based regulation of gas permeability by sealing a chamber with our uniaxial aligned Au‐LCON membrane and providing stimuli to “gate” the flow of test gases (**Figure** **3**a). We employ three gases in our measurements: apolar hydrogen (H_2_) and carbon dioxide (CO_2_), as well as polar ammonia (NH_3_). Heating the initially impermeable Au‐LCON membrane to over 40 °C induces LCON actuation and causes cracking in the Au layer, allowing gas molecules to penetrate the Au layer and diffuse into the LCON material. Once saturation is reached, the gas molecules are desorbed from the non‐coated side of the LCON. At this point, we can qualitatively detect the increased gas concentration outside the chamber using gas detectors for each type of gas. As shown in Figure 3b–d, we observe a similar trend for all controlled gas permeation, with our Au‐LCON membrane functioning well for both polar and apolar gases (Figure 3b–d). The crucial role of stimuli‐responsive anisotropic LCON deformation in this function is demonstrated by the lack of temperature‐responsive gas permeation control in an Au‐LCON membrane where the LCON material has no alignment, and thus does not actuate (Figure S4, Supporting Information).

We further demonstrate the switchable gas flow through our Au‐LCON membranes in the AcOH visualized gas permeation set‐up, illustrated previously in Figure 1g; heating the LCON above its actuation threshold activates deformation and forms cracks in the Au layer, allowing AcOH flow which leads to the initially purple litmus paper (Figure 3e‐i) turning red (Figure 3e‐ii). We also achieve passive localized gas permeability control by patterning the Au layer in our Au‐LCON membrane. A negative H‐form Au‐coated LCON membrane, similar to the negative of the pattern in Figure 1h, leaves a red H‐form on litmus paper due to the free flow of AcOH in the corresponding region; upon LCON actuation, the gas actively permeates across the entire membrane homogeneously, causing the H‐form to be erased as the entire litmus paper adopts a uniform red color (Figure 3f; Video S1, Supporting Information). This mechanism showcases the applicability of our Au‐LCON membranes as a chemical valve to control localized gas‐mediated reactions.

**Figure 4:**
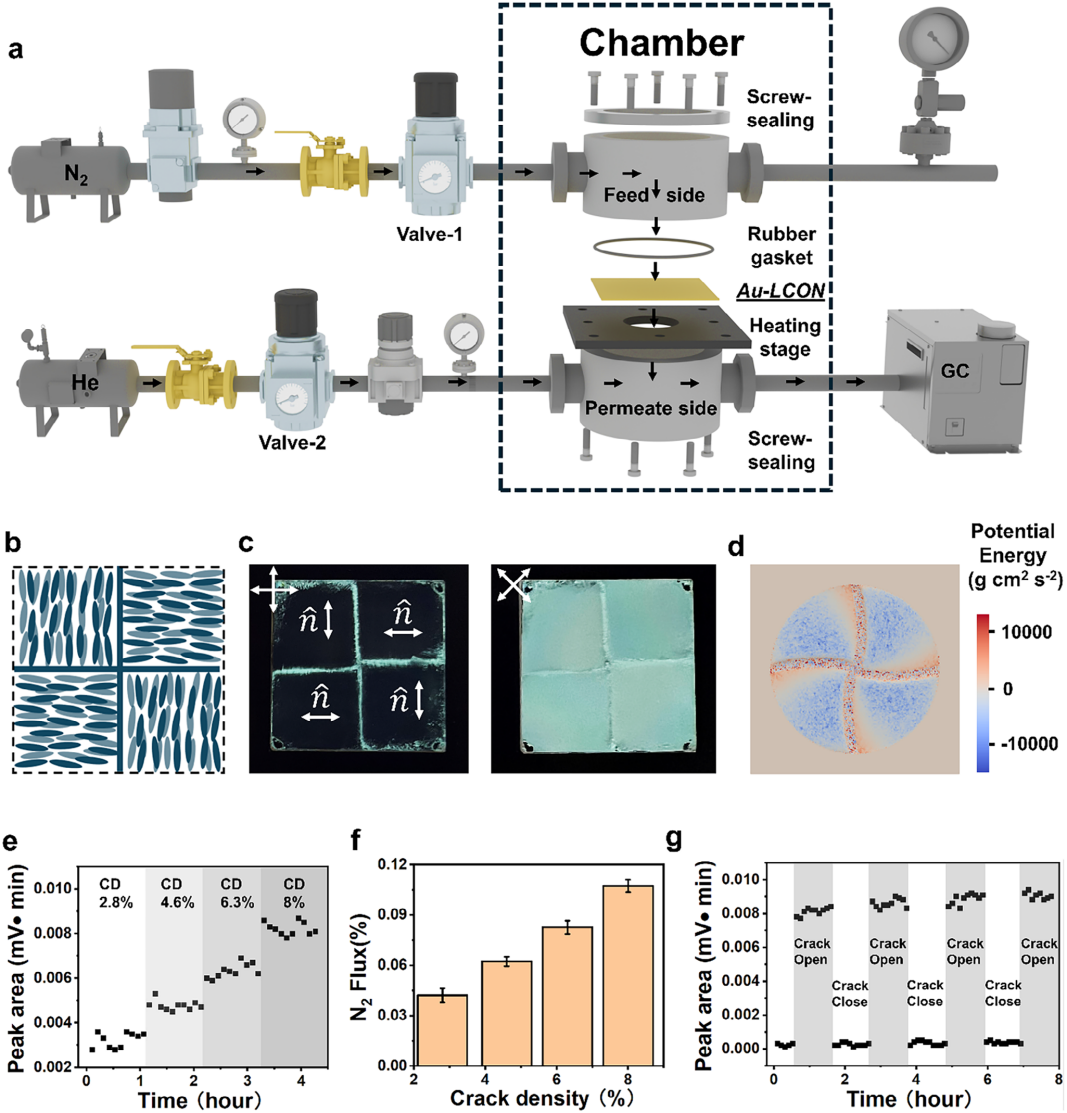
Dynamic and reliable gas permeability regulation in Au‐LCON membranes. a) Schematic illustration of the customized setup connected to GC; control valves and components are annotated. b) Schematic illustration of the quartet‐orthogonal‐alignment within the LCON of the adjusted membrane design. c) Crossed‐polarized optical images of the membrane with alternating orthogonal alignment; the polarizing directions of the crossed polarizers are marked and $\hat{n}$ denotes the director. d) FEM simulation results displaying the distribution of potential energy in the alternating orthogonal alignment membrane; the elastic potential energy of the film at its actuated state is represented in color in the embedded gradient scale. e) Graphical representation of the relationship between the amount of N_2_ permeating the Au‐LCON membrane and the crack density (CD) over time. f) Graphical representation of the relationship between the normalized flux of N_2_ and the CD. g) Graphical representation of the dynamic N_2_ permeation through the Au‐LCON membrane over time; the times when cracks in the Au‐layer are open or closed are marked.

We quantify the dynamics of the switchable gas permeability of our Au‐LCON membranes by measuring nitrogen gas (N_2_) permeation over time through gas chromatography (GC). A customized setup is shown in **Figure** **4**a; the Au‐LCON membrane is mounted on a temperature‐controlled plate, with N_2_ in the top chamber as the feed side and helium gas (He) in the bottom chamber as the permeated side. By adjusting the pressure on both sides, we achieve an equilibrium pressure to stabilize the membrane between the two chambers (Experimental section). N_2_ concentration on the permeated side is measured by recording the N_2_ levels over time, where the N_2_ peak areas in the GC results represent the integrated signal intensity corresponding to nitrogen concentration, enabling real‐time quantification of N_2_ permeation through the membrane.

However, since the membrane is confined at the peripheral boundary to seal both chambers, the shrinkage of the uniaxially aligned material causes it to undergo a high stretching force along the $\hat{n}$, resulting in a fracture of the membrane; this failure is analyzed through a finite element method (FEM) simulation, and computational results show potential energy accumulating at the boundary along the $\hat{n}$ of the membrane (Figure S5, Supporting Information). Therefore, to balance tension within the membrane and prevent fracture, we substitute the uniaxial alignment of the membrane with an alternating orthogonal alignment (Figure 4b; Figure S6, Supporting Information); we confirmed this alignment between two crossed polarizers (Figure 4c). The actuation simulation of a confined nematic polymeric LCON film with an alternative orthogonal alignment reveals that the highest stress for films with multiple alternating $\hat{n}$ is distributed around the polydomain intersections (Figure 4d). Hence, this $\hat{n}$ field design effectively reduces the mechanical stress around the LCON periphery, preventing membrane fracture.

Utilizing our improved $\hat{n}$ design, we analyze the gas permeability of our Au‐LCON membrane with different crack densities (CDs) (Figure 4e); higher CDs are observed to lead to increased gas permeation and vice versa. We also measure the corresponding normalized gas flux (Figure 4f), which we define as the amount of N_2_ passing through the Au‐LCON membrane divided by the amount of N_2_ passing through an LCON membrane without an Au coating (Figure S7, Supporting Information); based on this metric, our Au‐LCON membrane achieves 11% of the gas permeability of the pure LCON membrane (Figure 4f). By cycling the Au‐LCON membrane temperature above and below the threshold LCON actuation temperature, cracks in the Au layer can be opened and closed, allowing us to reversibly switch the membrane between permeable and impermeable states (Figure 4g). This demonstrates the reliability and flexibility for the permeability‐switching effect, which are crucial for practical application purposes.

## 3 Conclusion

In conclusion, we develop a stimuli‐responsive Au‐LCON bilayer membrane capable of on‐demand switchable gas permeability through the dynamic, thermally driven opening and closing of cracks in the impermeable Au layer; LCON actuation plays a crucial role in this function by inducing a stress mismatch at the Au and LCON interface to cause Au cracking. Once thermal stimuli are brought below the actuation threshold, the LCON relaxes to its initial geometry and closes cracks in the Au layer to restore the Au‐LCON membrane to its impermeable state. We utilize the switchable properties of our Au‐LCON membrane for real‐time gas flow monitoring in devices, acting as stimuli‐responsive chemical valves to regulate the progress and rate of vapor‐phase chemical reactions. We anticipate such intelligent Au‐LCON membranes functioning seamlessly without sensing equipment, relying on the intrinsic and tailorable responsiveness of the intelligent material, for feasible integration and precise operation in both micro‐scale and macro‐scale substance transport, gas exchange and molecular interactions. Furthermore, the chemical programmability of operating parameters and stimuli responsiveness, such as the capability of setting the LCON actuation threshold temperature, promises a major impact in controlled drug release applications. With the scalable synthesis of LCON membrane materials, its functionality can be extended to the remote regulation and quality control of food and pharmaceuticals, as well as to wearable health monitors that dynamically respond to the physiological changes of the user.

## 4 Experimental Section

(4‐(6‐(acryloyloxy)hexyloxy)phenyl‐4‐(6‐(acryloyloxy)hexyloxy)benzoate) (monomer **1**) is purchased from Daken Chemical. 2,2′‐(Ethylenedioxy) diethanethiol (molecule **2**), pentaerythritol tetrakis(3‐mercaptopropionate) (molecule **4**), dipropylamine (molecule **3**), tetrafunctional allyl ether crosslinker (molecule **5**), and acetic acid (AcOH) were obtained from Sigma‐Aldrich. Photoinitiator (molecule **6**) was obtained from BASF. Dichloromethane (DCM) was acquired from Biosolve. All reagents were used as received without further purification. The photo‐alignment agent SD1, was obtained from Nanjing JingCui Optical Technology Co., LTD, Nanjing, China.

### Preparation of the Uniaxial Au‐LCON Film

First, a mixture of diacrylate mesogen (monomer **1**), flexible dithiol spacer (molecule **2**), catalyst (molecule **3**), photoinitiator (molecule **6**), tetra‐functional thiol‐crosslinker (molecule **4**), and ene‐crosslinker (molecule **5**) was added to dichloromethane (DCM) to ensure mixing homogeneity. The resulting solution was then carefully poured into a polytetrafluoroethylene (PTFE) mold and left to react at room temperature for 12 h. Subsequently, the solvent was completely evaporated under vacuum conditions, and the film was gently removed from the mold, yielding a loosely crosslinked network film. To induce the alignment, the film was mechanically stretched along a single axis to 100% of its original length. Following this, the film was UV‐photopolymerized in N_2_ atmosphere at room temperature for 20 min, resulting in a uniaxially aligned LCON film. The Au layer was deposited onto the smooth side of the LCON using a sputter coater (Q150T Plus, Quorum). The thickness of the gold layer is determined by the sputtering current and time.

### Preparation of the Patterned LCON Film

The glass substrates were cleaned using acetone and isopropanol in an ultrasonic water bath for 20 min each. Afterward, the glasses were dried and subjected to UV‐ozone treatment for 20 min. The photo‐alignment agent SD1 was diluted 3 times with N, N‐Dimethylformamide (DMF) and then spin‐coated on the glass substrate. Subsequently, the coated glass substrate was baked at 100 °C for 10 min to completely evaporate the DMF to obtain a photo‐alignment layer on the glass substrate. A digital micro‐mirror‐based dynamic microlithography system was used to inscribe the alignment patterns on the coated substrate. To fabricate the LCON coating, the liquid crystal oligomers via a base‐catalyzed thiol‐acrylate Michael addition reaction was synthesized first. 1,4‐phenylene bis(4‐((6‐(acryloyloxy)hexyl)oxy)benzoate), dithiol, DPA, and photoinitiator were dissolved in the DCM solvent. The mixture was stirred overnight at room temperature. For the smaple used in Figure 2, after the Michael addition reaction was completed, the formed oligomers were spin‐coated on the patterned substrate, and then placed on a hot stage ≈55 °C to evaporate the solvent. For the sample shown in Figure 4, the mixture was injected into the liquid crystal cell to form a uniform film. Lastly, the LCON coating was obtained after polymerization using an EXFO Omnicure S2000 mercury lamp for 20 min.

### Characterizations

Differential scanning calorimetry (DSC) was conducted using a TA Instruments Q2000 DSC instrument from −40 to 100 °C at 10 °C min^−1^ with 10 mg product. Transition temperatures were determined from the third cycle. Dynamic mechanical thermal analysis (DMTA) was performed on TA Instruments Q800. The analysis was performed on a flat sample (5.1 × 5.0 × 0.6 mm^3^) in vertical tension mode. Measurements of storage modulus‐temperature and tanδ‐temperature curves were collected between −40 and 100 °C at a heating rate of 5 °C min^−1^ with 0.01 N preload force, 10 µm amplitude, and a 1 Hz oscillating frequency on a flat sample (5.1 × 5.0 × 0.6 mm^3^) in vertical tension mode. Actuation strain curves were collected between 25 and 75 °C at a heating rate of 5 °C min^−1^ in controlled force mode. Photographs and videos were taken with an Olympus OM‐D E‐M10 Mark III digital camera in manual mode. 3D microscopy images and corresponding 2D cross‐section profiles at various temperatures were captured using a Sensofar 3D optical profilometer equipped with a Linkam THMS600 hot stage for precise temperature control and atomic force microscopy (Cypher Environmental Scanner). Polarized optical microscopy images and reflective optical microscopy images were carried out on a Leica DM2700 M equipped with crossed polarizers and a Linkam THMS600 hot stage.

### Gas Permeability Test

To qualitatively test the gas permeability of the Au‐LCON membrane, a setup (Figure 3a) was designed and fabricated. The Au‐LCON membrane was carefully sealed over the central opening of a bottle cap using glue to ensure a gas‐tight fit. The Au‐coated side of the membrane was positioned facing inward toward the interior of the bottle, which contained a small amount of analyte gas, to prevent leakage through the LCON side. The bottle was then securely sealed with this membrane‐equipped cap. This assembly was then placed inside a conical flask, which was subsequently sealed with a rubber stopper to create an isolated environment. A probe from a gas detector (Shenzhen Honey Agile Technology Co., Ltd.) was then inserted through the rubber stopper into the conical flask to monitor the gas permeability.

To quantitatively and reversibly evaluate the tunable gas permeability of the membrane, we designed the experimental setup shown in Figure 4a. To ensure equilibration at the desired operational pressure difference between the feed and permeate sides, the valve was fine‐tuned accordingly. A supporting layer, composed of polycarbonate with a porous 10 µm membrane (Isopore), was placed above and below the membrane to prevent excessive expansion due to initial pressure instability during adjustment. For gas permeation analysis, a gas chromatography system (Trace 1300, Thermo Fisher Scientific) was integrated into the permeation flow path. The N₂ peak area in the GC results was used to quantitatively assess N₂ transport through the membrane.

### Visualized Gas Permeation Test

To visualize gas permeation, a patterned Au layer on the LCON film using masks was sputter coated. The patterned Au‐LCON membrane was then affixed over the central opening of a bottle cap, with a small amount of acetic acid vapor (AcOH) in the bottle. Once sealed, a piece of purple litmus paper was placed above the Au‐LCON membrane. Due to the patterned design, the AcOH vapor could permeate through the non‐Au‐coated regions of the LCON membrane, resulting in a visible pattern on the litmus paper where the gas passed through, turning those areas red. To demonstrate the dynamic gas permeability of the membrane, the sealed bottle was placed in an oven and the temperature was gradually increased. As the cracks were open under heating, the AcOH vapor was able to permeate across the entire surface of the Au‐LCON membrane, causing the purple litmus paper to turn entirely red.

### Finite Element

Actuation of a nematic elastomer thin film using a finite element method‐based elastoodynamics was modeled. The Hamiltonian described in that was used to describe actuation of a similar material was adopted. The geometry and obtaining the structured tetrahedral mesh were performed in Gmsh. To demonstrate the effect of alignment in the mesogenic units, two sets of director field designs, namely uniaxial and alternating orthogonal, were implemented. The distribution of potential energy for the case of uniaxial alignment is shown in Figure S5 (Supporting Information). The director field design for alternating orthogonal alignment, including the poly‐domains between the sections, is shown in Figure S6 (Supporting Information). Finally, the distribution of potential energy of the latter is shown in Figure 4.

## Conflict of Interest

The authors declare no conflict of interest.

## Supporting information



Supporting Information

Supporting Information

## Data Availability

The data that support the findings of this study are available from the corresponding author upon reasonable request.
