# Supplementary material for: Gating Gas Permeability Through Dynamic Cracking of Liquid Crystal Polymer Membranes
Source: Small. 2025 Jun 16;21(35):2503444. doi: 10.1002/smll.202503444 (PMC12410914; doi:10.1002/smll.202503444)
Supplement: Supplementary file 1 — Supporting Information [file SMLL-21-2503444-s001.docx]

Supporting Information

Gating gas permeability through dynamic cracking of liquid crystal polymer membranes

*Yuxin You, Youssef M. Golestani, Mert O. Astam, Danqing Liu**

Y. You, Y. M. Golestani, M. O. Astam, D. Liu

Human Interactive Materials (HIM)

Department of Chemical Engineering and Chemistry

Eindhoven University of Technology

Groene Loper 3, Eindhoven 5612AE, The Netherlands

E-mail: [danqing.liu@tue.nl](mailto:danqing.liu@tue.nl)

Y. You, Y. M. Golestani, M. O. Astam, D. Liu

Institute for Complex Molecular Systems (ICMS)

Eindhoven University of Technology

Groene Loper 3, Eindhoven 5612AE, The Netherlands

**The Supplementary Information includes:**

Figures S1-S7

Videos S1

We measure the storage modulus (**Figure S1a)** and the actuation strain (**Figure S1b)** of pure LCON at various temperatures using dynamic mechanical thermal analysis (DMTA). The maximum actuation strain of the LCON is higher than 40%.


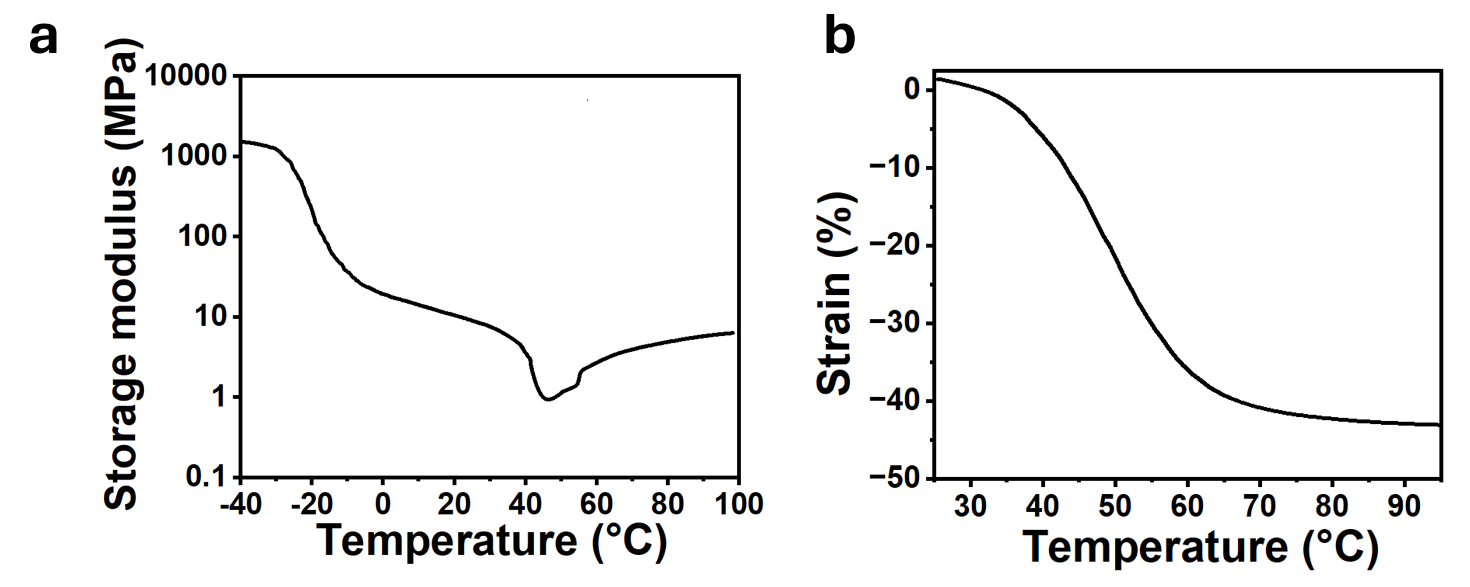


**Figure S1**. DMTA analysis of the LCONs demonstrating a) the storage modulus and b) the actuation strain.

We record the localized gas permeation using different Au-LCON membranes with varying Au-coated regions. During this process, AcOH gradually permeates, causing the litmus paper to change from entirely purple to region-specific red over 5 minutes. This gradual color change demonstrates the precise localization of gas permeation enabled by the selectively coated Au regions.


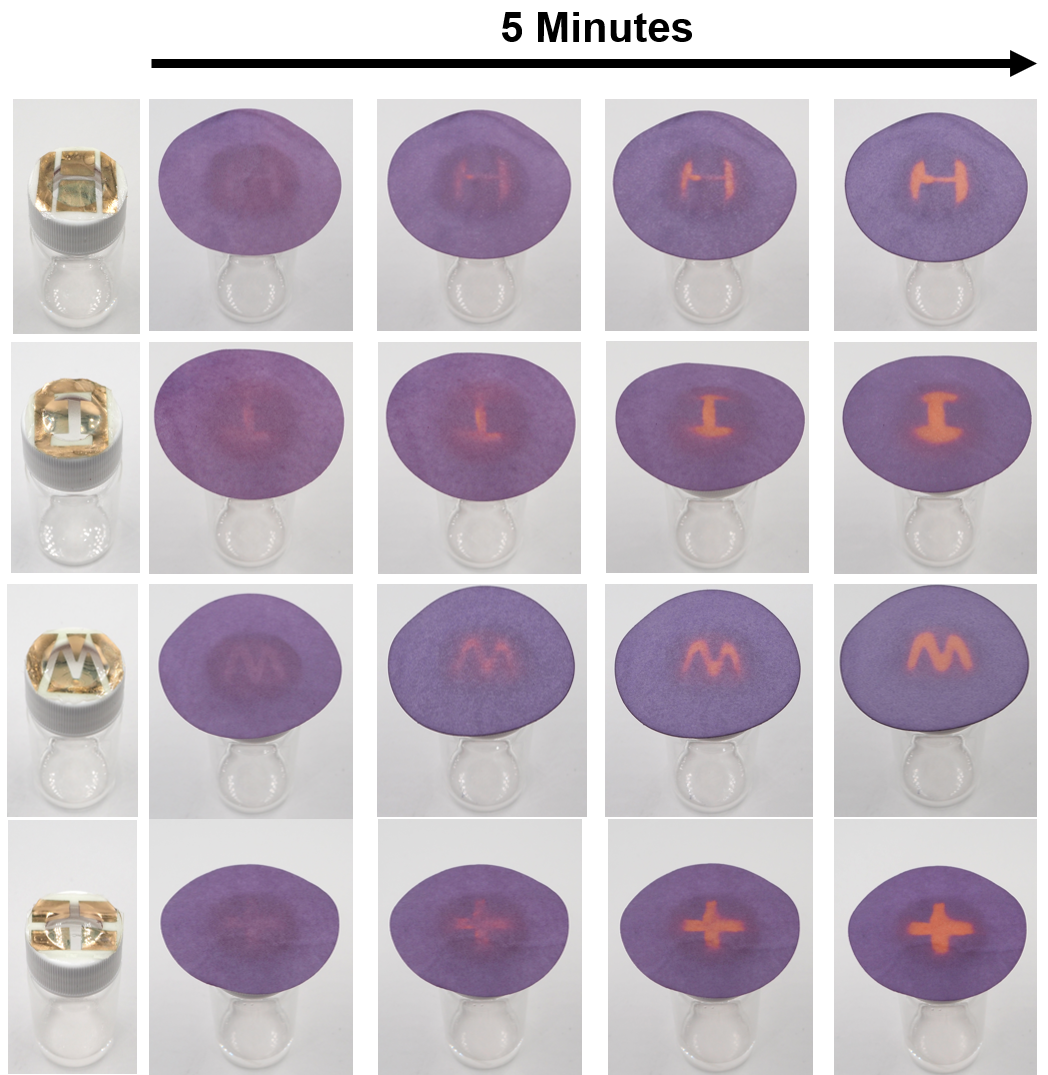


**Figure S2**. The localized permeation of AcOH leads to a region-specific red color changes on the litmus paper with different masks.

We define crack density as the ratio of the crack area to the total area in a given region using image analysis. We compare the crack density in the Au-LCON membranes with different thicknesses of Au layer, at strain of 30%, as shown in **Figure S3**. We find that the thicker the Au layer, the smaller the crack density. This is because a thicker Au layer can withstand higher stresses before cracking, distributing the strain more evenly and delaying crack initiation.


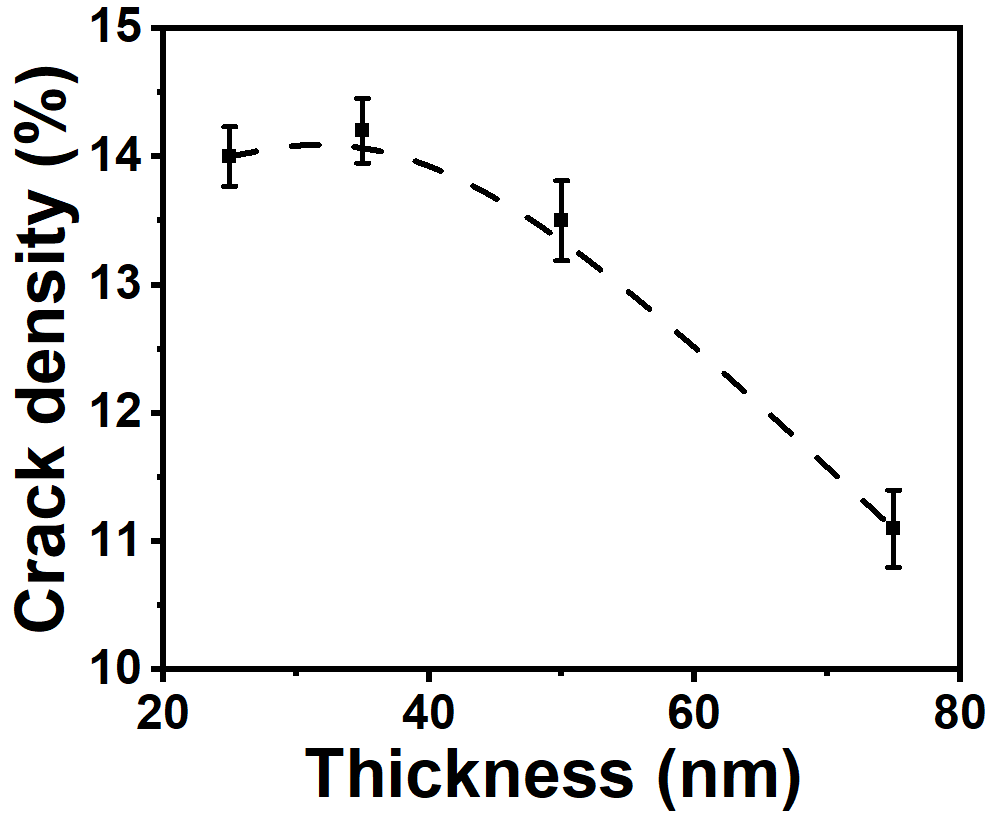


**Figure S3**. Relationship between the crack density and the thickness of Au.


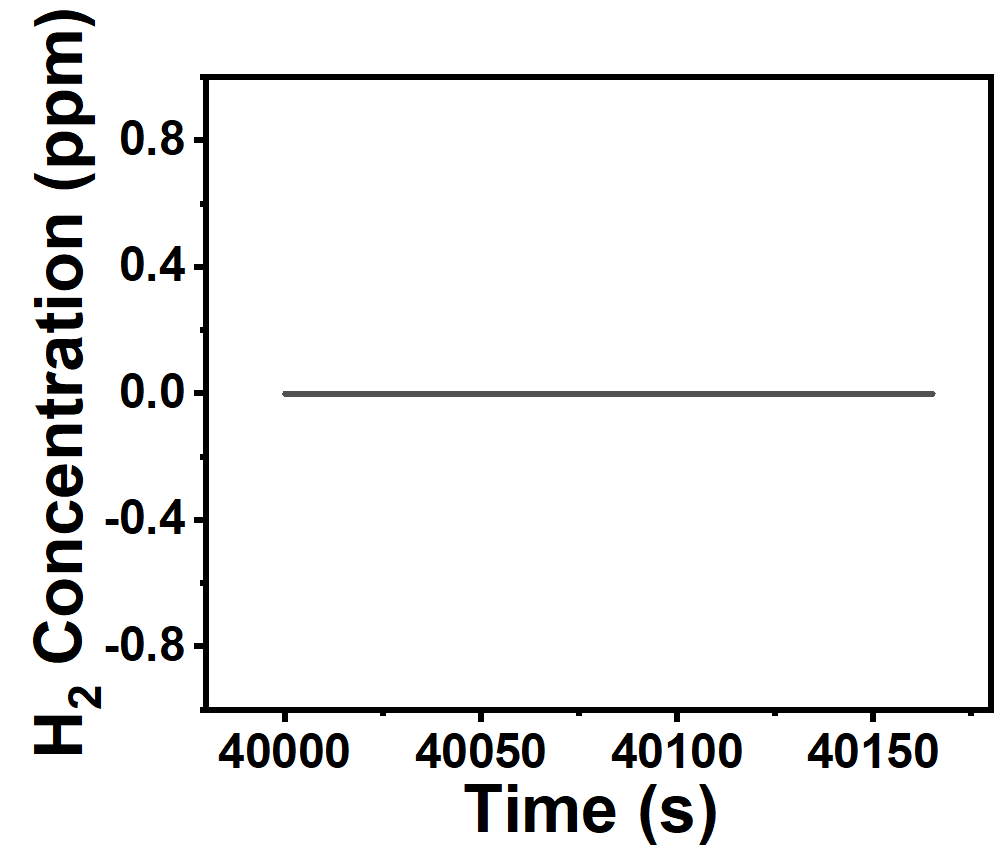


**Figure S4**. Measured concentration of H_2_ with an isotropic Au-LCON membrane.


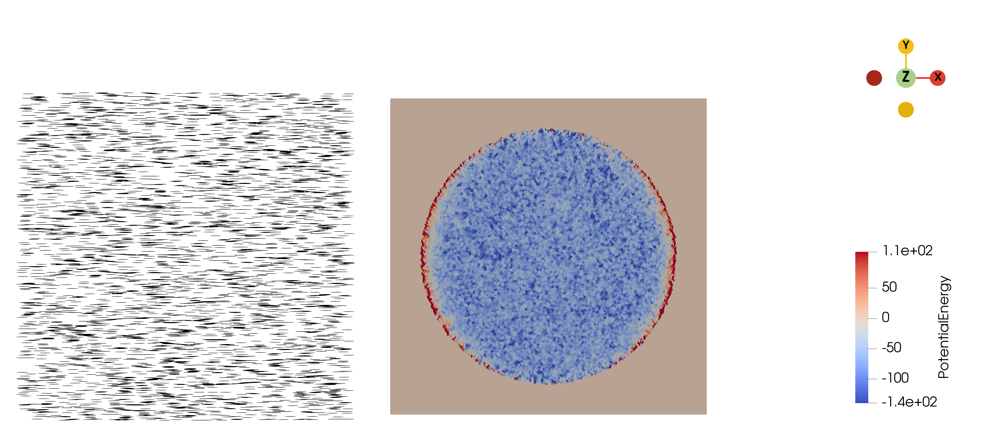


**Figure S5.** Director field design of uniaxial alignment (left), and the corresponding potential energy (right). The orange areas around the edges of the membrane are fixed as hard confinement. Upon actuation, the membrane exhibits significant potential energy along the uniaxial orientation, indicating that the membrane is prone to breakage at these two positions.


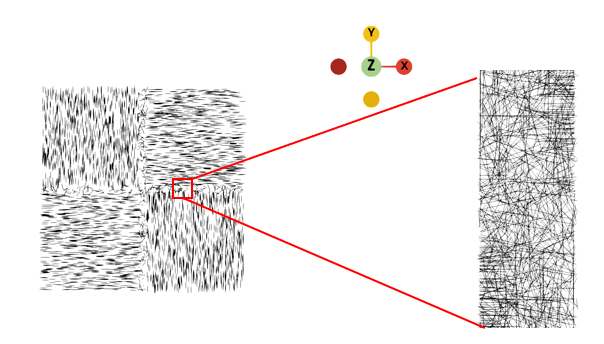


**Figure S6.** Director field design of the alternating orthogonal alignment in the simulation and the corresponding polydomain area.


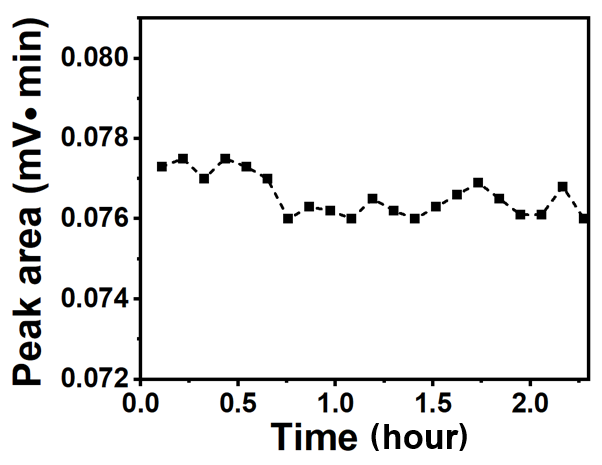


**Figure S7.** The amount of N_2_ passing through the pure LCON coating without Au layer measured by the customized setup connected to GC shown in Figure 4a.
